# Phosphate Acquisition and Virulence in Human Fungal Pathogens

**DOI:** 10.3390/microorganisms5030048

**Published:** 2017-08-22

**Authors:** Mélanie Ikeh, Yasmin Ahmed, Janet Quinn

**Affiliations:** 1 Oral and Craniofacial Biology, Center of Excellence in Oral Biology, LSUHSC School of Dentistry, New Orleans, LA 70119, USA; mikeh@lsuhsc.edu; 2 Institute for Cell and Molecular Biosciences, Faculty of Medical Sciences, Newcastle University, Newcastle upon Tyne NE2 4HH, UK; Y.Ahmed2@newcastle.ac.uk

**Keywords:** phosphate homeostasis, PHO pathway, Pho4 transcription factor, Pho80-Pho85 CDK, SPX domains, IP7 inositol polyphosphates, stress resistance, VTC complex, polyphosphate, fungal pathogenesis

## Abstract

The ability of pathogenic fungi to acquire essential macro and micronutrients during infection is a well-established virulence trait. Recent studies in the major human fungal pathogens *Candida albicans* and *Cryptococcus neoformans* have revealed that acquisition of the essential macronutrient, phosphate, is essential for virulence. The phosphate sensing and acquisition pathway in fungi, known as the PHO pathway, has been extensively characterized in the model yeast *Saccharomyces cerevisiae*. In this review, we highlight recent advances in phosphate sensing and signaling mechanisms, and use the *S. cerevisiae* PHO pathway as a platform from which to compare the phosphate acquisition and storage strategies employed by several human pathogenic fungi. We also explore the multi-layered roles of phosphate acquisition in promoting fungal stress resistance to pH, cationic, and oxidative stresses, and describe emerging roles for the phosphate storage molecule polyphosphate (polyP). Finally, we summarize the recent studies supporting the necessity of phosphate acquisition in mediating the virulence of human fungal pathogens, highlighting the concept that this requirement is intimately linked to promoting resistance to host-imposed stresses.

## 1. Introduction

Human fungal pathogens are a serious, but often neglected threat to human health. For example, it is estimated that fungal infections are responsible for over one million human deaths per annum [1]. Most of these fungi-attributed deaths are due to either *Cryptococcus*, *Candida*, or *Aspergillus* species which primarily affect immunocompromised patients. Of these species, the major fungal pathogens include *Cryptococcus neoformans*, *Candida albicans*, and *Aspergillus fumigatus*. *C. neoformans* and *A. fumigatus* are environmental fungi, naturally found in the soil and other ecological niches, and exposure to these pathogens occurs following inhalation of airborne spores. *C. neoformans* is the major causative agent of cryptococcal meningitis, which has a high incidence in HIV-1/AIDS populations, and leads to approximately 180,000 deaths each year [2]. *A. fumigatus* is responsible for over 200,000 cases of invasive aspergillosis each year in immunocompromised hosts, which is associated with alarming mortality rates of 50% at best [1]. This fungus also causes chronic pulmonary aspergillosis, a gradual destructive disease in the lung, which is estimated to affect 3 million people worldwide [1]. *C. albicans*, in contrast, is a common component of the gut microbiome, a frequent cause of oral thrush and vaginitis, and the most prevalent cause of life-threatening systemic fungal infections in immunocompromised patients. In fact, *Candida* spp. are the fourth leading cause of hospital-acquired bloodstream infections with a mortality rate above 40% [3]. In addition to *C. albicans*, the distantly related *Candida glabrata* species is also of concern due to increases in incidence [4]. Such increases may be due, in part, to the inherent resistance exhibited by this fungus towards several mainstream antifungal treatments [5]. 

It is well recognized that the ability of pathogenic fungi to acquire essential macro and micronutrients during infection is essential for virulence [6,7]. Phosphorus is an essential macronutrient for all organisms as it is found in nucleic acids, phospholipids, sugars, and proteins, and has vital biochemical roles in reactions that require the transfer of phosphate groups. Much is known regarding the phosphate acquisition strategies that promote the virulence of a number of bacterial pathogens [8]. In contrast, much less is known regarding the relationship between phosphate acquisition and virulence in pathogenic fungi. However, a number of recent studies have shown that perturbation of phosphate acquisition in two of the major human fungal pathogens, *Candida albicans* and *Cryptococcus neoformans*, attenuates virulence in a range of infection models. It is also emerging that phosphate acquisition and storage impact on stress resistance and metal homeostasis in fungi. In this review, we first of all summarize the well-characterized phosphate acquisition system in the model yeast *Saccharomyces cerevisiae*, as a platform from which to compare recent findings in phosphate acquisition and storage strategies in human pathogenic fungi. In addition, we describe the multifaceted roles of phosphate acquisition in promoting stress resistance and metal homeostasis, and review the cellular roles of the phosphate storage molecule polyphosphate (polyP). Finally, we summarized the current data supporting the necessity of phosphate acquisition in mediating the virulence of human fungal pathogens. 

## 2. The PHO System

The phosphate sensing and acquisition pathway, known as the PHO pathway, has been extensively characterized in the model yeast *S. cerevisiae* [9,10,11]. Hence, an overview of the *S. cerevisiae* PHO pathway will be given, followed by a comparison with what is known regarding related PHO pathways in human fungal pathogens.

### 2.1. S. cerevisiae

**Figure 1:**
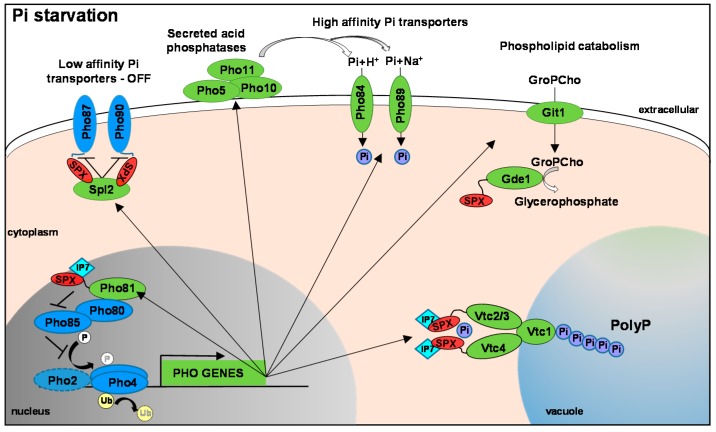
**The PHO Regulon.** This is based on the well characterised system in *S. cerevisiae* that is largely conserved in human fungal pathogens. In response to phosphate limitation the Pho4 transcription factor (TF) is dephosphorylated (and possibly deubiquitinylated). This allows for Pho4 nuclear accumulation and the Pho4-dependent expression of PHO genes (shown in green). In *S. cerevisiae* Pho4 works co-operatively with the Pho2 TF but homologues of this accessory factor have been shown to be (largely) dispensable for PHO gene expression in pathogenic fungi. Such PHO genes include secreted acid phosphatases, high affinity phosphate transporters and genes that can mobilise phosphate from phospholipids, which coordinate to increase phosphate acquisition. Genes involved in polyP synthesis are also induced, in addition to Spl2 which inhibits the activity of the low affinity phosphate transporters. Under phosphate replete conditions, Pho4 is phosphorylated by the Pho80-Pho85 CDK complex which results in exclusion from the nucleus. Following phosphate starvation the Pho81 CDK inhibitor prevents Pho85-mediated phosphorylation of Pho4. Pho81 is also a Pho4 regulated gene, and its induction positively feedbacks to Pho4. Several of these phosphate homeostasis proteins contain an SPX domain—indicated in red. These form a binding surface for IP7 inositol polyphosphate signalling molecules which likely coordinate distinct phosphate homeostasis mechanisms. Thus far IP7 binding to Pho81, Vtc3 and Vtc4 has been shown to modulate the function of these key proteins.

In response to phosphate limitation, activation of the PHO pathway in *S. cerevisiae* is initiated by the nuclear accumulation of the transcription factor Pho4, which leads to the induction of approximately twenty genes involved in phosphate acquisition, transport, and storage [12,13] (Figure 1). These include secreted acid phosphatases, such as Pho5, Pho10, and Pho11, which are localized to either the periplasmic space or the cell wall and scavenge phosphate from many different substrates [14]. Such secreted acid phosphatases work in concert with the high affinity phosphate transporters, Pho84 and Pho89, which are also induced under low phosphate conditions. Pho84 is an H^+^/Pi symporter with a pH optima of 4.4, whereas Pho89 is a Na^+^/Pi symporter with a pH optima of 9.5, thus ensuring that phosphate can be acquired over a wide pH range [15]. In addition, a negative regulator of the Pho87 and Pho90 low affinity phosphate transporters, Spl2, is also induced [12,13]. A complex feedback loop within the PHO pathway exists that modulates the activity of the low and high phosphate transporters depending on phosphate ability [16]. Following phosphate starvation, activation of Pho4 triggers both a negative feedback loop (by inducing the expression of the Pho84 high affinity transporter) which brings phosphate into the cell and inactivates Pho4, and a positive feedback loop (involving the Pho4-dependent upregulation of *SPL2*) which negatively regulates the Pho87 and Pho90 low affinity transporters, thus reducing phosphate uptake leading to pathway activation. In cells lacking Pho84, the negative feedback loop is lost, but the Spl2-mediated inhibition of the low affinity transporters is still in place which causes additional reductions in intracellular phosphate that further drives activation of the pathway. Thus, in *pho84Δ* cells, the PHO pathway is constitutively on but phosphate uptake is negligible, and this underlies phenotypes associated with loss of Pho84 including an inability of *pho84Δ* cells to survive on phosphate limiting media and the constitutive expression of secreted acid phosphatase activity [16]. Gene expression profiling also revealed that yeast cells scavenge phosphate from phospholipids due to the upregulation of the *GIT1* and *GDE1* genes [13,17]. Deacylation of the major phospholipid, phosphatidylcholine, by phospholipases generates glycerophosphocholine (GroPCho), which is transported into the cell by the Git1 permease [17]. The GroPCho is subsequently hydrolyzed by the Gde1 phosphodiesterase, to choline and glycerolphosphate which provides a phosphate source [18]. The PHO pathway also regulates the induction of genes involved in the synthesis of the phosphate storage molecule polyP. In yeast, polyP is mainly stored in the vacuole and its synthesis is regulated by the Vacuolar Transporter Chaperone (Vtc) complex. This comprises of Vtc1, Vtc2, Vtc3, and Vtc4, with Vtc4 being the catalytic subunit [19]. All four Vtc complex genes are upregulated following phosphate starvation in a Pho4 dependent manner [12,13].

The key regulator of the PHO regulon, the Pho4 basic helix-loop-helix transcription factor, activates induction of the genes described above co-operatively with the Pho2 transcription factor [13] (Figure 1). Under phosphate rich conditions, Pho4 is phosphorylated on specific serine residues by the Pho80-Pho85 cyclin-dependent kinase (CDK) complex [20,21] which prevents its accumulation in the nucleus [22]. Specifically, five Serine-Proline (SP1 to SP4 and SP6) sites are phosphorylated, with each site playing a distinct role in the regulation of Pho4. Phosphorylation at SP2 and SP3 ensures Pho4 nuclear export, while phosphorylation at SP4 promotes its import, and phosphorylation at SP6 prevents interaction with the Pho2 co-activator [23]. The inhibitory effect of Pho80-Pho85 on Pho4 activity following phosphate starvation is prevented by the action of the CDK inhibitor, Pho81. Pho81, by inhibiting the Pho80-Pho85 CDK complex, leads to the dephosphorylation and nuclear accumulation of Pho4 and the induction of the PHO regulon [20,21]. In addition to phosphorylation, a recent proteome-wide screen of yeast deubiquitylating enzymes revealed that ubiquitination of Pho4 may also negatively regulate the ability of this transcription factor to accumulate in the nucleus and induce the PHO regulon [24]. A summary of the key regulators of the PHO pathway, together with their orthologs in pathogenic fungi are given in Table 1.

Recent advances in elucidating the mechanisms controlling phosphate homeostasis have highlighted the role of the so-called SPX domain (for an excellent review see [10]). This was originally identified in yeast and is named after Suppressor of Yeast *gpa1* (Sgy1), the Phosphatase 81 (Pho81), and the human Xenotropic and Polytropic Retrovirus receptor 1 (Xpr1). A number of the key proteins involved in phosphate homeostasis described above contain a SPX domain, including the Pho81 CDK inhibitor, the Vtc2, Vtc3, and Vtc4 proteins needed for polyphosphate synthesis, the Pho87 and Pho90 low affinity phosphate transporters, and the Gde1 GroPCho phosphodiesterase (Figure 1) [10]. The crystal structure of the SPX domain from a number of different proteins, including the yeast Vtc4 polyphosphate polymerase, has recently been published [25]. This comprises of a unique three helix bundle that shares no major structural homology with proteins of known function. At the N-terminus of the SPX helical bundle is a basic binding surface which has a high affinity for IP6 and IP7 inositol polyphosphate (IP) signaling molecules [25]. As IP7 levels change depending on phosphate availability within the growth environment [25,26,27], an exciting concept is emerging that SPX domains function as cellular receptors for IP signaling molecules that co-ordinate multiple roles in phosphate sensing and signaling. In this regard, as Pho81 has an SPX domain, it is relevant that almost a decade ago IP7 was shown to stimulate Pho81-dependent inhibition of Pho80-Pho85 [26]. IP7 was found to interact non-covalently with the Pho80-Pho85-Pho81 complex, inducing additional interactions between Pho81 and Pho80-Pho85, which ultimately prevented access to, and thus phosphorylation of, Pho4 [28]. It has also been shown that the low affinity phosphate transporters Pho87 and Pho90, described above, interact with their negative regulator Spl2, via their SPX domains, resulting in a downregulation of phosphate uptake [29]. Therefore, based on recent findings it is reasonable to speculate that IP7 occupancy of the SPX domains within Pho87/Pho90 regulate binding to Spl2. Indeed, following on from the crystallization of the Vtc4 SPX domain, it was shown that mutation of conserved residues within the SPX domain in either Vtc3 or Vtc4 prevented IP7 binding in vitro and polyP synthesis in vivo [25]. This is consistent with earlier genetic studies which showed that mutants unable to produce IP signaling molecules had undetectable levels of polyP [27]. Taken together, these studies illustrate that IP7 sensing by SPX domain-containing proteins play a major role in coordinating distinct phosphate homeostasis mechanisms in *S. cerevisiae*. 

### 2.2. C. glabrata

Of the human fungal pathogens considered in this review, the commensal pathogen *C. glabrata* is most closely related to the model yeast *S. cerevisiae.* Consistent with this, studies revealed a high degree of conservation of the PHO pathway in *C. glabrata* with that in *S. cerevisiae* [32]. Mutational analysis revealed that lack of *PHO4* or *PHO81* in *C. glabrata* cells prevented the production of secreted acid phosphatases and the induction of *PHO84* and *GIT1* under phosphate limiting conditions, whereas deletion of *PHO80* resulted in the constitutive activation of the pathway [32]. Thus, as in *S. cerevisiae*, Pho80-Pho85-Pho81 mediated regulation of Pho4 seemingly forms the core of the PHO pathway in *C. glabrata*. However, in contrast to *S. cerevisiae*, Pho2 is largely dispensable for Pho4-dependent gene expression in *C. glabrata*. In addition, ectopic expression of *C. glabrata PHO4* in *S. cerevisiae* bypassed the need for Pho2 in PHO pathway activation [32]. A recent phylogenetic survey revealed that the reduced dependence on Pho2 for PHO gene expression evolved in *C. glabrata* and closely related species [33]. This study also revealed that bypassing the need for Pho2 leads to a dramatic expansion of Pho4 target genes in *C. glabrata* compared to that in *S. cerevisiae* [33]. Remarkably, only 16 of the 79 genes directly induced by Pho4 in *C. glabrata* are involved in maintaining phosphate homeostasis, including phosphate transporters such as *PHO84*, several acid phosphatases, the *VTC1-4* genes involved in polyP synthesis, and *GIT1* and *GDE1* involved in obtaining phosphate from phospholipids [33]. The remaining genes are implicated in other virulence-associated attributes including stress resistance, cell wall synthesis, and adhesion [33]. Thus, it is possible that the functional expansion of Pho4-targets in *C. glabrata* reflects an adaptation of this pathogen to particular environmental niches within the human host. 

### 2.3. C. albicans

Recent studies have uncovered a similar PHO pathway in the important fungal pathogen *C. albicans*, with the analogous Pho4 transcription factor being essential for growth under phosphate limiting conditions [34,35]. Similar to *S. cerevisiae* Pho4, *C. albicans* Pho4 accumulates in the nucleus following phosphate starvation and regulates the induction of a number of phosphate responsive-genes [36]. These include the high affinity phosphate transporter *PHO84*, several acid phosphatase genes, and the *VTC1* and *VTC3* genes required for polyP synthesis [36]. Consistent with this, *pho4Δ* cells had lower intracellular phosphate levels, no detectable polyP, and drastically reduced secreted acid phosphatase activity [36]. Phospholipids are also likely important sources of phosphate in *C. albicans*, as Pho4 regulates the induction of the *GIT1-3* genes, and the *GDE1* gene [36,37,38]. Similar to that seen in *S. cerevisiae* [16], the Pho4 target gene, Pho84, is essential for growth on phosphate limiting media, with loss of *PHO84* driving the constitutive upregulation of secreted acid phosphatase activity [39]. This suggests that a similar feedback loop that regulates the switching of phosphate transporters in *S. cerevisiae* is also operational in *C. albicans*. However, as reported in *C. glabrata*, the homologue of Pho2, Grf10, in *C. albicans* is dispensable for phosphate homeostasis [34]. Moreover, as in *C. glabrata*, [33] loss of Pho4 in *C. albicans* impacts on the transcription of numerous genes not involved in phosphate homeostasis [36]. Although chromatin immunoprecipitation studies are needed to ascertain which of these genes are directly regulated by Pho4, it is interesting to speculate that such gene expansion is due to the lack of dependence on the co-activator Pho2, as recently reported in *C. glabrata* [33]. Moreover, the concept that such gene expansion could facilitate adaptation to the host environment is supported by the fact that the *C. albicans* PHO pathway is also required for resistance to a diverse range of other stresses in addition to phosphate starvation [36,40,41]. Such stress-protective roles of Pho4 are considered later in this review. 

How *C. albicans* Pho4 is regulated in response to phosphate limitation has yet to be ascertained. Sequence homology between the *S. cerevisiae* and *C. albicans* Pho4 transcription factors is largely restricted to the C-terminal DNA-binding domain [40] with the N-terminal regulatory regions exhibiting significant divergence. In particular, key phosphorylation sites implicated in Pho4 regulation in *S. cerevisiae* largely do not align with the *C. albicans* Pho4 sequence. However, the *PHO81* gene encoding the Pho80-Pho85 CDK inhibitor is induced following phosphate limitation in *C. albicans* [36], suggesting a possible conservation of the positive feedback loop that exists in *S. cerevisiae*. In addition, *C. albicans* Pho85 has been shown to complement loss of *PHO85* in *S. cerevisiae* [42], and depletion of *PHO85* in *C. albicans* results in increased levels of intracellular phosphate, consistent with this kinase negatively regulating Pho4 [39]. As discussed in the following section, *C. albicans* Pho4 is also critical for gene expression and survival under alkaline pH conditions which, as previously reported in *S. cerevisiae* [43], triggers a phosphate starvation response. 

Recent work also indicates possible transcription-independent roles of the PHO pathway in *C. albicans.* For example, there is emerging evidence from *C. albicans* and other fungal species that intracellular phosphate levels may play key roles in regulating metal homeostasis [36], and this is discussed in more detail later in this review. In addition, a recent forward genetic screen in *C. albicans* uncovered a previously uncharacterized role for phosphate homeostasis in regulating the target of rapamycin (TOR) pathway—the central growth control module of the cell [39]. This connection is conserved in *S. cerevisiae* [39]. Specifically, the high affinity phosphate transporter Pho84, was shown to be important for the activity of Target of Rapamycin Complex 1 (TORC1) in both *C. albicans* and *S. cerevisiae* in a mechanism involving the TORC1-stimulating GTPase, Gtr1. Thus, the TOR1 pathway monitors phosphate availability in addition to carbon and nitrogen sources.

### 2.4. C. neoformans

In the human pathogen *C. neoformans*, the study of the PHO pathway was hampered due to the difficulty in identifying candidate orthologs of Pho4 [44]. It was only by screening mutants generated by T-DNA insertion mutagenesis, or the recently generated transcription factor deletion collection [45], for strains defective in secreted phosphatase production, that led to the identification of this transcription factor [46,47]. Indeed, *C. neoformans* Pho4 displays a higher degree of sequence homology to *C. albicans* Pho4, compared to the analogous transcription factor in *S. cerevisiae* [47]. Nonetheless, *C. neoformans* Pho4 accumulates in the nucleus following phosphate starvation and is essential for growth, and the production of secreted acid phosphatases, under phosphate limiting conditions [47]. Moreover, there is large degree of overlap in the core PHO regulon in *C. neoformans* to that seen in *S. cerevisiae* and *C. albicans*. These include the high affinity phosphate transporters *PHO84*, *PHO840*, and *PHO89*, the *VTC1* and *VTC4* genes involved in polyP synthesis, the secreted acid phosphatase *AHP1* (*PHO2*), and the *GIT1* and *GDE1* genes necessary for phospholipid catabolism [44,46,47]. Chromatin immunoprecipitation studies demonstrated the direct binding of Pho4 to many of these genes [46]. However, as in *C. glabrata* and *C. albicans*, *C. neoformans* Pho4 likely regulates additional gene targets in addition to the core PHO gene set. Pho4 was found to bind to over 110 genes in *C. neoformans* and loss of Pho4 impacted on the transcription of genes not directly implicated in phosphate homeostasis [46]. However, further experimentation is needed to explore the physiological relevance of this.

With regard to Pho4 regulation in *C. neoformans*, homologues of the key regulators, Pho80, Pho85, and Pho81 were identified by BLAST searches [46]. Subsequently, cells lacking *PHO80* or harboring mutations in *PHO85* were found to result in constitutive activation of the PHO pathway, as measured by secreted phosphatase activity, and *C. neoformans PHO85* could complement loss of *S. cerevisiae PHO85* [46]. Moreover, drug-induced inhibition of Pho85 activity stimulated secreted acid phosphatase activity under phosphate replete conditions, in a mechanism requiring Pho4 [47]. In contrast, secreted acid phosphatase activity was inhibited in cells lacking the CDK inhibitor Pho81 [46]. Thus, taken together these data indicate that, as in *S. cerevisiae*, Pho85-Pho80 negatively regulate Pho4 in *C. neoformans*, and that Pho81 counteracts this repression in phosphate limiting environments. Notably, however, differences in phosphate homeostasis mechanisms were also uncovered in this fungal pathogen. For example, in addition to *PHO81*, a number of other regulatory proteins were induced in *C. neoformans* following phosphate starvation including the CDK and cyclin genes, *PHO85* and *PHO80* [46]. Moreover, Pho4 was found to bind to its own promoter and regulate its own expression; such autoregulation of Pho4 has not been described previously [46]. In addition, there is evidence of functional redundancy between high affinity phosphate transporters in *C. neoformans* that has not been described previously. Deletion of all three phosphate transporters (Pho84, Pho840, Pho89) is needed to impair *C. neoformans* growth under phosphate starvation conditions [44], whereas loss of Pho84 alone inhibits growth of both *S. cerevisiae* and *C. albicans* upon phosphate starvation. In addition, whilst deletion of the three high affinity phosphate transporters in *C. neoformans* drastically impairs polyP levels [44], *pho4Δ* cells display wild-type levels of polyP in this fungal pathogen [47]. This contrasts with the lack of polyP in other *pho4Δ* mutants [12,36]. However, consistent with a global role of Pi acquisition in survival of alkaline pH stress, as reported in other yeasts, *C. neoformans* Pho4 is also vital for survival under alkaline conditions and regulates the induction of phosphate acquisition genes in response to this stress [47]. This is considered in more detail in the following section, together with a summary of other stress resistance roles associated with phosphate acquisition in *C. neoformans* [44,47].

### 2.5. A. fumigatus

There is considerably less information on the PHO pathway in *A. fumigatus* than in the fungal pathogens described above. However, deletion of the *A. fumigatus PHO80* orthologue *phoB*^PHO80^ resulted in cells that displayed constitutive secreted acid phosphatase activity, increased phosphate uptake, and increased levels of vacuolar polyP [48]. Consistent with these phenotypes, transcript profiling studies identified a number of the core PHO genes that were constitutively expressed in Δ*phoB^PHO80^* cells, including the high affinity phosphate transporters *phoD*^PHO84^ and *phoE*^PHO89^, a number of acid and alkaline phosphatases, the CDK inhibitor *phoC*^PHO81^, and the orthologue of Vtc4, *vtcD*^VTC4^ [48]. Collectively this data is consistent with *phoB*^PHO80^ having a similar negative role in regulating activation of PHO pathway in *A. fumigatus* as established in *S. cerevisiae*. Interestingly however, *A. fumigatus* cells lacking *phoD*^PHO84^ did not display the phenotypes associated with *PHO84* loss in *S. cerevisiae* or *C. albicans* [48]. This is reminiscent of that reported in *C. neoformans* [44], in which functional redundancy exists between the high affinity phosphate transporters.

A summary of the key differences in PHO pathway regulation in human pathogenic fungi compared to *S. cerevisiae* is presented in Table 1. 

## 3. Phosphate Acquisition and Stress Resistance

**Figure 2:**
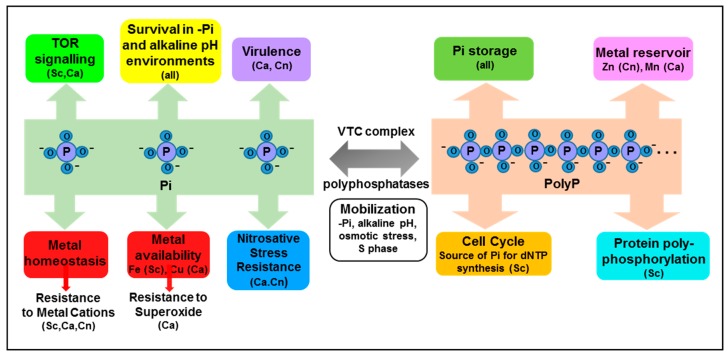
**Emerging roles of intracellular Pi and polyP.** Phosphate plays well-established roles in many biomolecules and biochemical processes, and is stored in the vacuole as polyP. However additional roles of inorganic phosphate (Pi) and polyP are emerging in fungi, and are summarised above. See text for details. Abbreviations: Sc; *S. cerevisiae*, Ca; *C. albicans*, Cn; *C. neoformans*.

A number of studies have revealed the importance of intracellular phosphate levels in mediating resistance to seemingly divergent stress conditions. These are described below and summarized in Figure 2. 

### 3.1. Alkaline pH Resistance

Transcript profiling studies in *S. cerevisiae* illustrated that in response to alkaline pH, the PHO regulon was rapidly induced [43]. Subsequently, it has been established in a number of fungi that switching to growth under alkaline conditions triggers a phosphate starvation response. According, in *S. cerevisiae*, *C. albicans*, and *C. neoformans*, the Pho4 transcription factor accumulates in the nucleus upon the switch to alkaline growth and there is also a rapid mobilization of phosphate from vacuolar polyP phosphate reserves [36,47,49]. Such Pho4-mediated responses are clearly important, as cells lacking this transcription factor are acutely sensitive to growth under alkaline pH conditions [36,47,50]. As both low affinity Pi transport by Pho87 and Pho90 [51], and high affinity transport by Pho84 [52], relies on H^+^/Pi symport, the phosphate starvation state triggered by alkaline pH is most likely due to disruption of the proton gradient across the plasma membrane under pH stress. Consistent with this, *C. neoformans* cells grown under alkaline pH conditions in the presence of radioactive-labelled orthophosphate displayed significantly impaired uptake of phosphate compared to cells grown under acidic conditions [47]. Moreover, consistent with phosphate uptake being prevented, addition of excess phosphate to the media does not rescue the alkaline stress sensitive phenotypes of *pho4Δ* cells [47]. Presumably in wild-type cells, the Pho4-mediated expression of the high affinity Na^+^/Pi Pho89 symporter, possibly in conjunction with the Git1-Gde1 phosphate acquisition system, promotes survival. Significantly, as discussed later, many of the virulence defects associated with loss of Pho4 in *C. neoformans* were found to be related to impaired growth in alkaline host environments [47]. Related to this, in *C. albicans*, the impaired serum-induced filamentation exhibited by *pho4Δ* cells was found to be due to the alkaline pH of serum [36].

### 3.2. Cation Resistance

Evidence is also emerging that alterations in phosphate levels, which is the most abundant cellular anion, have wide-ranging effects on the homeostasis of biologically important metal cations [53]. In *S. cerevisiae*, artificially increasing intracellular levels of phosphate (by inactivating the Pho80 negative regulator of Pho4), has profound effects on metal cation accumulation and toxicity [53]. Dramatic increases in cellular levels of calcium and sodium occur in *pho80Δ* cells, together with an increased toxicity of these and many other metal cations including manganese, cobalt, zinc, and copper [53]. In this regard, it is interesting that *A. fumigatus* cells lacking the orthologous *PhoD*^PHO80^ gene also display increased sensitivity to calcium [48]. The huge increases in cellular calcium and sodium influx observed in *S. cerevisiae pho80Δ* cells could potentially counter the negative charge imposed by increased phosphate. Increased intracellular phosphate levels also trigger a characteristic iron starvation response in *S. cerevisiae* [53], indicating that phosphate binding to iron decreases the bioavailability of this essential metal. In *C. neoformans*, depletion of intracellular phosphate levels (via deletion of *PHO84*, *PHO840*, and *PHO89*) also impacted on metal homeostasis, resulting in a significant accumulation of sodium cations as well as increases in zinc and iron levels [44]. Such cells also displayed increased sensitivity to calcium, sodium, and manganese cations [44]. Thus, studies in *S. cerevisiae* and *C. neoformans* illustrate that both increases and decreases in intracellular phosphate levels can stimulate increases in metal cation levels and impair the cellular resistance to such metals. In *C. albicans*, deletion of Pho4 significantly reduced intracellular phosphate levels and resulted in impaired resistance to a multitude of both metal and non-metal cations [36,41]. The fact that *pho4Δ* cells were also sensitive to non-metal cations such as spermidine, indicates that the protective role of intracellular phosphate is not restricted to metals, and that a key function is in maintaining a charge balance in the cell. This is further supported by the observation that although sensitive to many metal cations, the cellular levels of such metals in *pho4Δ* cells are similar to that seen in wild-type cells [36]. The exception to this was manganese, whose levels where were dramatically reduced in *pho4Δ* cells [36]. *C. neoformans* cells lacking Pho4 were also notably sensitive to calcium cations indicating possible conservation of phosphate acquisition in promoting cation resistance [47]. In support of this, the sensitivity of *C. neoformans* to CaCl_2_ could be rescued by supplementation of the media with phosphate [47]. 

### 3.3. Resistance to Oxidative and Nitrosative Stresses

In addition to promoting resistance to alkaline pH and cationic stress, Pho4 has recently been implicated in oxidative and nitrosative stress resistance in *C. albicans* [36,41], and nitrosative stress resistance in *C. neoformans* [47]. Of the oxidative stress-sensitive phenotypes reported for *C. albicans pho4Δ* cells, the most striking is that towards the superoxide stress-generating compound menadione [36,41]. However, Pho4 does not accumulate in the nucleus in response to superoxide stress and does not appear to directly regulate the induction of superoxide stress-protective genes [36]. Instead, it appears that defects in copper bioavailability underlie the acute sensitivity of *pho4Δ* cells to superoxide stress as this results in a significant reduction of the activity of the copper-dependent superoxide dismutase (Sod1) enzyme [36]. Evidence in support of this comes from the observation that both Sod1 activity and superoxide stress resistance in *pho4Δ* cells are completely rescued by supplementation of the growth medium with copper [36]. Moreover, *C. albicans* cells lacking Pho4, despite containing wild-type levels of intracellular copper, display significant resistance to this potentially toxic metal [34,36], which may be due to increased expression of the copper sequestering metallothionein gene, *CRD2* [36]. Thus, the current model is that impaired bioavailability of copper, possibly by increase in copper metallothionein levels, results in impaired Sod1 activity that culminates in impaired superoxide stress resistance in *pho4Δ* cells. Interesting, such roles of phosphate homeostasis regulating copper availability do not appear to be conserved in *C. neoformans* [47], as Pho4 is dispensable for superoxide stress resistance in these fungi. In contrast, the role for Pho4 in promoting nitrosative stress resistance is seemingly conserved between diverse fungal pathogens [41,47], however the mechanism underlying this is currently unknown.

To summarize, in addition to regulating phosphate acquisition in phosphate limiting environments, the Pho4 transcription factor mounts a similar transcriptional response to alkaline pH stress and is essential for survival under such conditions. It is also worth highlighting that the gene-expansion of Pho4 target genes in pathogenic fungi compared to that in the model yeast *S. cerevisiae* [33], may also place other cellular processes under Pho4-mediated transcriptional regulation. However, loss of Pho4 also triggers phenotypes that are likely independent of direct transcriptional regulation. Most notably, modulation of intracellular phosphate levels by either preventing or hyperactivating Pho4, has diverse impacts on metal cation toxicity, accumulation, and bioavailability which can influence diverse processes such as oxidative stress resistance. Moreover, as metal acquisition and detoxification strategies are vital for fungal survival at the host/pathogen interface [54], the importance of intracellular phosphate levels in metal homeostasis merits further investigation.

## 4. Polyphosphate

Mobilization of phosphate from the phosphate storage molecule polyP is one of the first responses evoked in fungi in response to phosphate limiting conditions. In addition, a conserved aspect of the PHO regulon between fungal species is the upregulation of genes involved in the synthesis of polyP. As described above in Section 2.1, polyP synthesis in fungal cells is mediated by the VTC complex, a vacuolar membrane protein assembly composed of the polyP synthetase Vtc4, Vtc1 and either Vtc2 or Vtc3 (reviewed in [55]). In *S. cerevisiae*, *C. albicans*, and *C. neoformans*, cells lacking Vtc4 have no detectable polyP [12,36,44]. In the opposite reaction, mobilization of polyP following phosphate starvation is facilitated predominantly by two polyphosphatases Ppn1 and Ppx1, although other enzymes with polyphosphatase activity exist (reviewed in [55]). Consistent with this role, *C. neoformans* cells lacking orthologues of Ppn1 and Ppx1 (Epp1 and Xpp1, respectively) contained increased levels of polyP [44]. 

In addition to phosphate storage, polyP has been implicated in an array of processes in prokaryotic cells (reviewed in [56,57]), which may relate to recent findings that polyP functions as a protein chaperone in bacteria [58]. Despite current intense interest in this molecule [59], much less is known about polyP function is eukaryotic microbes. There is some evidence that in lower eukaryotes polyP function mediates stress adaptation and osmoregulation (reviewed in [60]). For example, exposure of *Trypanosoma cruzi* to hypo-osmotic stress results in the rapid mobilization of polyP, whereas hyper-osmotic stress triggers an increase in poly-P levels [61]. Here, we provide an update on polyP function in *S. cerevisiae* and human pathogenic fungi which is summarized in Figure 2. 

In *C. albicans*, it was revealed that in addition to alkaline pH conditions, phosphate is mobilized from polyP following both hypo-osmotic stress and hyper-osmotic stress. However, the physiological significance of this is not clear, as polyP is seemingly dispensable in mediating resistance to such stresses in *C. albicans* [36]. In fact, the only function attributed to polyP thus far in *C. albicans* is manganese resistance, with polyP playing an important role in the sequestration of this particular metal [36]. Interestingly, *C. neoformans* cells either lacking polyP, or in which polyP mobilization was impaired, both displayed impaired tolerance to zinc, suggesting a role for polyP in zinc homeostasis in this fungal pathogen [44]. Such roles in metal homeostasis are reminiscent of the well-established role of polyP in sequestering diverse cations in bacterial cells (reviewed in [62]). Intriguingly, in a recent study, polyP containing structures were located on the surface of *C. neoformans* cells [63]. In this regard, as polyP is known to induce blood clotting [64], this may underlie previous findings that wild-type but not *vtc4Δ C. neoformans* cells could increase the rate of blood clotting [44].

Evidence is emerging from studies in the model yeast *S. cerevisiae*, that polyP plays a role in cell cycle progression in fungi. PolyP levels were found to drop when *S. cerevisiae* cells go through S-phase, and this is possibly mediated by Ppn1, as the expression levels of this polyphosphatase coincided with the observed decreases in polyP [65]. Moreover, *vtc4Δ* cells displayed a delay in cell cycle progression that was more pronounced in Pi limiting media, and this was due to such cells taking significantly longer to replicate their DNA [65]. Consistent with this, under phosphate limiting conditions, whilst wild-type cells significantly increase dNTP synthesis at the G1/S transition in readiness for DNA replication, this was not seen in cells lacking polyP or unable to mobilize polyP [65]. Thus, taken together, these data illustrate that one of the roles played by polyP in *S. cerevisiae*, and conceivably in other fungal cells, is the provision of enough phosphate for the upsurge of dNTP synthesis at the end of G1 phase to allow for efficient DNA replication. In an in vitro competition experiment between wild-type and *vtc4Δ* cells, cells lacking polyP were consistently underrepresented in the mixed population, reflecting the reduced fitness of the *S. cerevisiae vtc4Δ* mutant [65]. Interestingly, similar findings were reported with *C. albicans pho4Δ* cells, which were replaced by wild-type cells when grown in mixed cultures in vitro [41]. Moreover, these findings were replicated in an in vivo competition assay in which *pho4Δ* cells were unable to colonize the murine gut when inoculated simultaneously with wild-type cells [41]. Based on the recent findings in *S. cerevisiae* [65], it is interesting to speculate that the reduced fitness of *C. albicans pho4Δ* cells may be due to a lack of polyP, resulting in delays in DNA replication. This is further supported by the observation that genes upregulated in *C. albicans pho4Δ* cells, even under phosphate replete conditions, cluster to the functional categories of “DNA Metabolism”, “DNA Repair and Response to DNA Damage”, and “Cell Cycle” [36]. Furthermore, a *C. neoformans* triple high affinity phosphate transporter mutant, which lacked polyP, displayed an enlarged cell type indicative of possible problems with cell cycle control and delayed cell division [44]. Collectively, these findings support the notion that polyP regulates cell cycle progression in several fungi.

A further exciting role of polyP, recently identified in *S. cerevisiae*, is the covalent attachment of polyP to target proteins as a novel post-translational modification [66]. This modification was shown to occur on lysine residues within a polyacidic PASK domain found in both the Nsr1 (nuclear signal recognition 1) protein and its interacting partner Top1 (topoisomerase) [66]. Such polyphosphorylation did not occur in cells lacking Vtc4, and the exopolyphosphatase Ppx1 was able to remove such groups, thus uncovering a previously unrecognized signaling role. Importantly, polyphosphorylation has obvious physiological functions as this disrupted the Nsr1/Top1 interaction and impaired Top1 enzymatic activity [66]. Thus, it will be exciting to extend the findings in *S. cerevisiae* and explore this newfound signaling role of polyP in protein polyphosphorylation in pathogenic fungi. 

## 5. Phosphate Acquisition and Virulence

A number of recent studies have shown that perturbation of phosphate acquisition in both *C. albicans* and *C. neoformans* impacts on virulence in a range of infection models. The first clue that phosphate acquisition could be important for *C. albicans* virulence came from the observation that the Pho4-target gene, *PHO84*, which encodes a high affinity phosphate transporter, is upregulated in a number of infection models [67,68,69]. This was further supported by studies which revealed that the Pho4-dependent phosphate acquisition genes, the acid phosphatase Pho100 and the glycerophosphocholine permease Git3, are required for virulence in murine models of systemic infection [37,70]. More recently, *C. albicans* cells lacking the major regulator of the PHO regulon, Pho4, have been shown to display attenuated virulence in both systemic and commensal animal models of infection [36,41]. Moreover, cells lacking *PHO4* were acutely sensitive to macrophage-mediated killing in one study [36], but not in a separate study [41]. The reasons for this are unclear but may reflect significant differences in study design and execution. Whether the impaired ability to survive phagocytosis reported in [36] is due to defects in phosphate acquisition or the importance of phosphate homeostasis in promoting resistance to the cationic and superoxide stresses encountered following macrophage uptake is not clear. However, Sod1, which requires Pho4 function for activity, is important for *C. albicans* survival following phagocytosis [71], as are key regulators of *C. albicans* cationic stress resistance [72], thus is it temping to speculate that the stress-protective functions of Pho4 promote survival following phagocytosis. 

Interestingly, a recent study described two clinical *C. albicans* isolates that displayed filamentous growth on media lacking phosphate which was not recapitulated in standard clinical reference or laboratory strains [35]. Moreover, in a mouse model of infection in which phosphate is limiting, such strains were hypervirulent, and this trait could be reversed upon oral administration of phosphate [35]. This indicates that these clinical isolates display enhanced phosphate-dependent virulence. What could be the underlying mechanism? It was suggested that this could be due to the enhanced filamentation of such clinical isolates under phosphate limiting conditions. Evidence in support of this came from the observation that under low phosphate conditions loss of Pho4 promoted both filamentation and virulence of *C. albicans* in the *Caenorhabditis elegans* nematode infection model [35]. As filamentation is a virulence determinant in *C. elegans* [73], this supports the concept that the enhanced filamentation underlies such hypervirulence of *pho4Δ* cells [35]. However, no obvious loss-of-function substitutions were observed in Pho4 from such clinical isolates [35]. It is also noteworthy that enhanced secreted acid phosphatase levels were observed in the clinical isolates, suggesting perhaps that the pathway was hyper-activated rather than impaired, which may in turn result in enhanced virulence. A better understanding of the deregulation of the PHO pathway in the clinical isolates isolated in this study is needed to comprehend the phosphate-dependent hypervirulent phenotypes. Indeed, Pho4 was found to be important for *C. albicans* virulence in the *C. elegans* infection model under phosphate replete conditions [36], thus indicating that in phosphate-rich environments Pho4 is critical for virulence. As above, whether this is due to the low intracellular levels of phosphate that persist in *pho4Δ* cells in phosphate replete media [36], or other stress-resistant roles of Pho4 remains to be determined.

As in *C. albicans*, the indication that phosphate acquisition may be important for virulence in *C. neoformans* came from in vivo transcript profiling studies following macrophage uptake, which revealed that the high affinity phosphate transporter *PHO84* showed the greatest level of induction (sixteen-fold) of all induced transporters [74]. Following this, it was found that a *C. neoformans* triple mutant lacking all three high affinity high phosphate transporters (Pho84, Pho840, and Pho89), in addition to impaired growth on phosphate lacking media also resulted in reduced formation of capsule and melanin, both of which are virulence traits in this fungal pathogen [44]. Consistent with this, in a mouse inhalation model, mice infected with the triple phosphate transporter mutant survived significantly longer than mice infected with wild-type cells, despite the fact that the fungal load in lungs and brain was comparable to that of wild-type cells [44]. Moreover, this triple mutant was unable to replicate following phagocytosis by macrophages and showed impaired survival compared to wild-type cells [44]. Most recently, an extensive study on the impact of Pho4 loss on *C. neoformans* virulence has been published [47]. In a mouse inhalation (pulmonary) model of infection, the *pho4Δ* mutant displayed significantly impaired virulence, showing massively reduced fungal burdens in the brain, whereas much more minimal effects were seen with lung fungal burdens. Intriguingly, the virulence defect of *pho4Δ* cells was even more pronounced in a mouse dissemination model of infection. The growth rate of *pho4Δ* cells in lung and brain tissues, and in the blood, was compared to wild-type and reconstituted strains during infection. Remarkably, this revealed that the *pho4Δ* mutant was never cultured from the blood [47]. Subsequent investigations revealed that the reduced dissemination of *pho4Δ* cells to the brain is due to the vital role of Pho4 in promoting growth in the alkaline pH environment of the blood [47]. This explains why no CFUs were detected in the blood from animals infected with the *pho4Δ* strain. As discussed above, alkaline pH mimics a phosphate limiting environment, and thus *pho4Δ*-deficicient cells cannot thrive in such environments [47]. In this regard, it is interesting to note that the *C. neoformans* triple phosphate transporter mutant could disseminate to the brain [44]. These cells did display some growth, albeit impaired, on alkaline growth media [44], which might underlie why this mutant, but not that lacking Pho4, could at least partially survive blood exposure to disseminate to the brain. In a separate study, the Pho4-dependent secreted acid phosphatase, Ahp1, was found to be produced in cryptococci following incubation with monocytes and to be important for *C. neoformans* virulence in both *Galleria mellonella* and mouse infections models, further supporting the role of phosphate acquisition in the virulence of this fungal pathogen [75]. It is also noteworthy, due to the emerging key roles of inositol polyphosphate IP7 molecules in phosphate sensing and signaling, that IP7 is also required for *C. neoformans* pathogenicity [76,77]. However, whether such IP7-mediated impaired virulence in *C. neoformans* is due to defects in phosphate homeostasis remains to be addressed.

Whilst there is strong evidence that the PHO pathway is essential for both *C. albicans* and *C. neoformans* virulence, evidence is lacking that this is the case in *A. fumigatus*. Cells lacking either *phoB*^PHO80^ or *phoD*^PHO84^ did not display attenuated virulence in a murine low dose model of invasive aspergillosis [48]. However, loss of *phoB*^PHO80^ hyper-activates the PHO pathway, and evidence was presented suggesting that *phoD*^PHO84^ functions redundantly with other phosphate transporters in *A. fumigatus* [48]. Therefore, virulence testing of a strain with a defective PHO pathway is needed before the role of phosphate acquisition in *A. fumigatus* virulence can be concluded. 

## 6. Concluding Remarks

This is an exciting time in the phosphate homeostasis field. Recent notable findings include new insight into how cytosolic phosphate levels are sensed by the binding of inositol polyphosphate signaling molecules to SPX domains, located in a multitude of phosphate homeostasis regulators. Furthermore, new roles for the phosphate storage molecule, polyP, are emerging, in addition to the newly identified post-translation modification of protein ‘polyphosphorylation’. Studies in fungi are also highlighting the importance of intracellular phosphate homeostasis in TOR signaling and mediating resistance to a range of diverse stress conditions. It is also now clear that phosphate homeostasis is an important virulence trait in human pathogenic fungi. Significantly, a recent study demonstrated the potential benefit of targeting phosphate acquisition strategies in the treatment of systemic mycoses, in that a small molecule inhibitor of Pho84 was found to potentiate the activity of the antifungals amphotericin B and micafungin [39]. An important challenge ahead is to connect the new findings from model and pathogenic fungi to drill down into the mechanisms linking phosphate acquisition with fungal virulence to deliver new antifungal treatments. 

## Figures and Tables

**Table 1:** Key Regulators of the *S. cerevisiae* PHO Regulon and their Orthologues in Human Fungal Pathogens.

Gene	Function in *S. cerevisiae*	*C. glabrata* ^1^ Orthologue and % Identity	*C. albicans* ^1^ Orthologue and % Identity	*C. neoformans* ^2^ Orthologue and % Identity	*A. fumigatus* ^3^ Orthologue and % Identity	Key Differences Compared to *S. cerevisiae*
*PHO4*	Basic helix-loop-helix transcription factor; activates transcription cooperatively with Pho2p in response to phosphate limitation. Required for polyP production	*PHO4* (CAGL0D05170g) 35%	*PHO4* (C4_05680W_A) 29%	*PHO4/HLH3* (CNAG_06751) 29%	Afu5g04190 33%	Significantly larger than Sc Pho4; homology mainly restricted to DNA binding domain. *CnPHO4* displays auto-regulation. No impact on polyP levels in Cn *pho4Δ* cells.
*PHO2*	Homeodomain transcription factor; activates transcription cooperatively with Pho4p in response to phosphate limitation.	*PHO2* (CAGL0L07436g) 39%	*GRF10* (C5_05080W_A) 34%		Afu4g10220 25%	Role of Pho2 largely restricted to Sc. No clear orthologue in Cn.
*PHO80*	Cyclin component of the Pho80-Pho85 CDK complex. See Pho85 description.	*PHO80* (CAGL0E02541g) 53%	*PHO80* (C6_03810W_A) 49%	*PHO80* (CNAG_01922) 34%	*phoB* (Afu1g07070) 48%	*PHO80* is induced in Cn following Pi starvation.
*PHO85*	Cyclin dependent kinase of the Pho80-Pho85 CDK complex. Phosphorylates Pho4 under phosphate replete conditions preventing nuclear accumulation.	*PHO85* (CAGL0L12474g) 89%	*PHO85* (C1_04520C_A) 67%	*CMGC/CDK/CDK5* (CNAG_08022) 60%	*phoA* Afu5g04130 68%	*PHO85* is induced in Cn following Pi starvation.
*PHO81*	CDK inhibitor that counteracts Pho85-Pho80 activity in low phosphate conditions. Induced following Pi starvation.	*PHO81* (CAGL0L06622g) 42%	*PHO81* (CR_00590W_A) 37%	*PHO81* (CNAG_02541) 29%	*phoC* (Afu4g06020) 30%	
*PHO84*	High affinity phosphate transporter. Loss of *PHO84* results in constitutive activation of the PHO pathway but negligible phosphate uptake.	*PHO84* (CAGL0B02475g) 77%	*PHO84* (C1_11480W_A) 61%	*PHO84* (CNAG_02777) 47%	*phoD* *Afu2g10690* 55%	Cn *PHO84* displays functional redundancy with *PHO840* and *PHO89.* Possible functional redundancy between Af *phoD* and other Pi transporters.

^1^ Sequences obtained from the *Candida* Genome Database. ^2^ Sequences obtained from the *C. neoformans* H99 strain annotated database [30]. ^3^ Sequences obtained from the Aspergillus Genome Database. Pairwise comparison with the Sc protein was performed using EMBOSS Needle protein alignment (http://www.ebi.ac.uk/Tools/psa/emboss_needle/) [31] and percentage identity calculated as the number of conserved residues/Sc protein length. Abbreviations: Sc, *S. cerevisiae*; Cg, *C. glabrata*; Ca, *C. albicans*; Cn, *C. neoformans*; Af, *A. fumigatus*.
